# Strong Foam-like Composites from Highly Mesoporous Wood and Metal-Organic Frameworks for Efficient CO_2_ Capture

**DOI:** 10.1021/acsami.1c06637

**Published:** 2021-06-15

**Authors:** Shennan Wang, Cheng Wang, Qi Zhou

**Affiliations:** † Division of Glycoscience, Department of Chemistry, School of Engineering Sciences in Chemistry, Biotechnology and Health, KTH Royal Institute of Technology, AlbaNova University Centre, Stockholm SE-106 91, Sweden; ‡ Advanced Fibro-Science, Kyoto Institute of Technology, Kyoto 606-8585, Japan; § Wallenberg Wood Science Center, Department of Fibre and Polymer Technology, KTH Royal Institute of Technology, Stockholm SE-100 44, Sweden

**Keywords:** MOFs, mesoporous wood template, composites, CO_2_ capture, mechanical properties

## Abstract

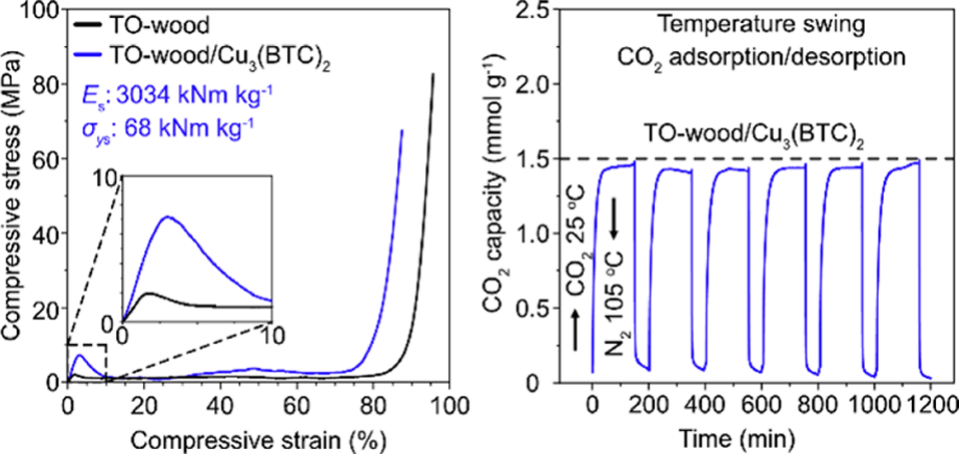

Mechanical stability and multicycle durability are essential for emerging solid sorbents to maintain an efficient CO_2_ adsorption capacity and reduce cost. In this work, a strong foam-like composite is developed as a CO_2_ sorbent by the in situ growth of thermally stable and microporous metal-organic frameworks (MOFs) in a mesoporous cellulose template derived from balsa wood, which is delignified by using sodium chlorite and further functionalized by 2,2,6,6-tetramethylpiperidine-1-oxyl (TEMPO)-mediated oxidation. The surface carboxyl groups in the TEMPO-oxidized wood template (TO-wood) facilitate the coordination of the cellulose network with multivalent metal ions and thus enable the nucleation and in situ growth of MOFs including copper benzene-1,3,5-tricarboxylate [Cu_3_(BTC)_2_], zinc 2-methylimidazolate, and aluminum benzene-1,3,5-tricarboxylate. The TO-wood/Cu_3_(BTC)_2_ composite shows a high specific surface area of 471 m^2^ g^–1^ and a high CO_2_ adsorption capacity of 1.46 mmol g^–1^ at 25 °C and atmospheric pressure. It also demonstrates high durability during the temperature swing cyclic CO_2_ adsorption/desorption test. In addition, the TO-wood/Cu_3_(BTC)_2_ composite is lightweight but exceptionally strong with a specific elastic modulus of 3034 kN m kg^–1^ and a specific yield strength of 68 kN m kg^–1^ under the compression test. The strong and durable TO-wood/MOF composites can potentially be used as a solid sorbent for CO_2_ capture, and their application can possibly be extended to environmental remediation, gas separation and purification, insulation, and catalysis.

## 1 Introduction

CO_2_ capture technologies are of great importance to mitigate the greenhouse gas emission and reduce its environmental impact.^1^ Enormous efforts have been previously made to develop solid CO_2_ sorbents with high energy efficiency and multicycle durability by using porous materials including zeolite,^2^ silica,^3^ and activated carbon,^4,5^ or amine-based sorbents with CO_2_-reactive polyethyleneimine (PEI),^6^ 3-(triethoxysilyl)propylamine (APTES),^7^ and ethylenediamine.^8,9^ Recently, metal-organic frameworks (MOFs) have attracted much attention for applications in CO_2_ capture,^10−12^ catalysis,^13−15^ and sensing,^16^ owing to their favorable large surface area and tunable micro-/mesopore structure. Mechanical integrity and strength are essential for the practical application of solid sorbents to avoid pulverization and thus overcome high pressure drop and poor mass transfer.^17^ To this end, monolithic MOF-based CO_2_ sorbents have been prepared through strategies such as stepwise gelation of MOFs,^18^ 3D printing,^19^ and in situ growth of MOFs in preformed inorganic network including porous carbon,^20^ macro-/mesoporous silica,^21^ and graphene hydrogel.^22^ For instance, graphene/zeolitic imidazolate framework-8 (ZIF-8) hybrid aerogel showed an elastic modulus of 280 kPa and a maximum strength of 16 kPa under compressive deformation.^22^ Utilizing clay and poly(vinyl alcohol) (PVA) as the binder and plasticizer enabled the 3D printing of a cobalt-based MOF (UTSA-16) into a channeled monolithic structure, which showed an elastic modulus of 25 MPa and a compressive strength of 0.55 MPa at a density of 1659 kg m^–3^.^19^

In addition, deposition, encapsulation, or in situ synthesis of MOF particles such as zirconium terephthalate-based MOF (UiO-66), copper-based MOF (MOF-199), and ZIF-8 in natural wood was developed. The corresponding wood/MOF composites were demonstrated to have high removal efficiency for organic pollutants,^23^ superior selectivity toward CO_2_ adsorption against N_2_,^24^ and good antibacterial activities.^25^ Furthermore, carbonization of wood-/cobalt-based MOF composites produced a high-power 3D monolithic reactor for improved mass transfer and CO conversion during Fischer–Tropsch synthesis.^26^ These composites have combined the functionalities of MOFs and mechanical robustness of wood. Particularly, beech wood with in situ synthesized ZIF-8 showed a high compressive strength of 100 MPa, which outperformed polymer-based MOF composites.^24^ However, insufficient coordination sites in cell lumen surfaces of natural wood resulted in the low loading of MOFs around 2 wt %, limiting wood/MOF composites to be used for the large capacity adsorption of CO_2_.^23,24^ The binding and adhesion of MOFs to the cellulosic substrate can be enhanced by introducing surface functional groups such as carboxyl groups. High loading of MOFs (>30 wt %) has been previously reported when using 2,2,6,6-tetramethylpiperidine-1-oxyl (TEMPO)-oxidized cellulose nanofibers (TO-CNFs) with surface carboxyl groups as the substrate for the interfacial synthesis of MOFs toward pollution remediation,^27^ thermal insulation,^28^ energy storage,^29^ and volatile organic compound separation.^30^ However, the potential of using surface carboxylated CNFs for developing strong and durable sorbents for CO_2_ capture has not yet been explored.

In our previous study, a hierarchical 3D network of cellulose microfibrils was prepared from wood through a top-down delignification process, followed by TEMPO-mediated oxidation at neutral conditions.^31^ This TEMPO-oxidized wood (TO-wood) showed a highly mesoporous cell wall structure with fibrillated but naturally aligned cellulose microfibrils and demonstrated high mechanical performance. Herein, we report a facile approach to fabricate foam-like cellulose/MOF composites with prominent mechanical properties, good thermal stability, and high CO_2_ adsorption capacity using TO-wood structure as the template. The carboxyl groups in the fibrillated cell wall of TO-wood facilitated interfacial coordination to copper (Cu^2+^)-, zinc (Zn^2+^)-, and aluminum (Al^3+^)-based MOFs and promoted their growth in situ, thus increasing the loading of MOFs. The specific surface area, CO_2_ adsorption capacities, multicycle durability under temperature swing adsorption, and compressive mechanical properties of the TO-wood/MOF composites were studied to demonstrate their potential application as a strong and durable sorbent for efficient CO_2_ capture.

## 2 Experimental Section

### 2.1 Chemicals and Materials

Balsa wood (*Ochroma pyramidale*) was purchased from Materials AB, Sweden. Cu(NO_3_)_2_·(H_2_O)_3_, Al(NO_3_)_3_, Zn(NO_3_)_2_·(H_2_O)_6_, benzene-1,3,5-tricarboxylic acid (H_3_BTC), 2-methylimidazole, TEMPO, sodium chlorite, and sodium hypochlorite were purchased from Sigma-Aldrich, Germany and used as received.

### 2.2 Preparation of the TO-Wood Template

**Scheme 1:**
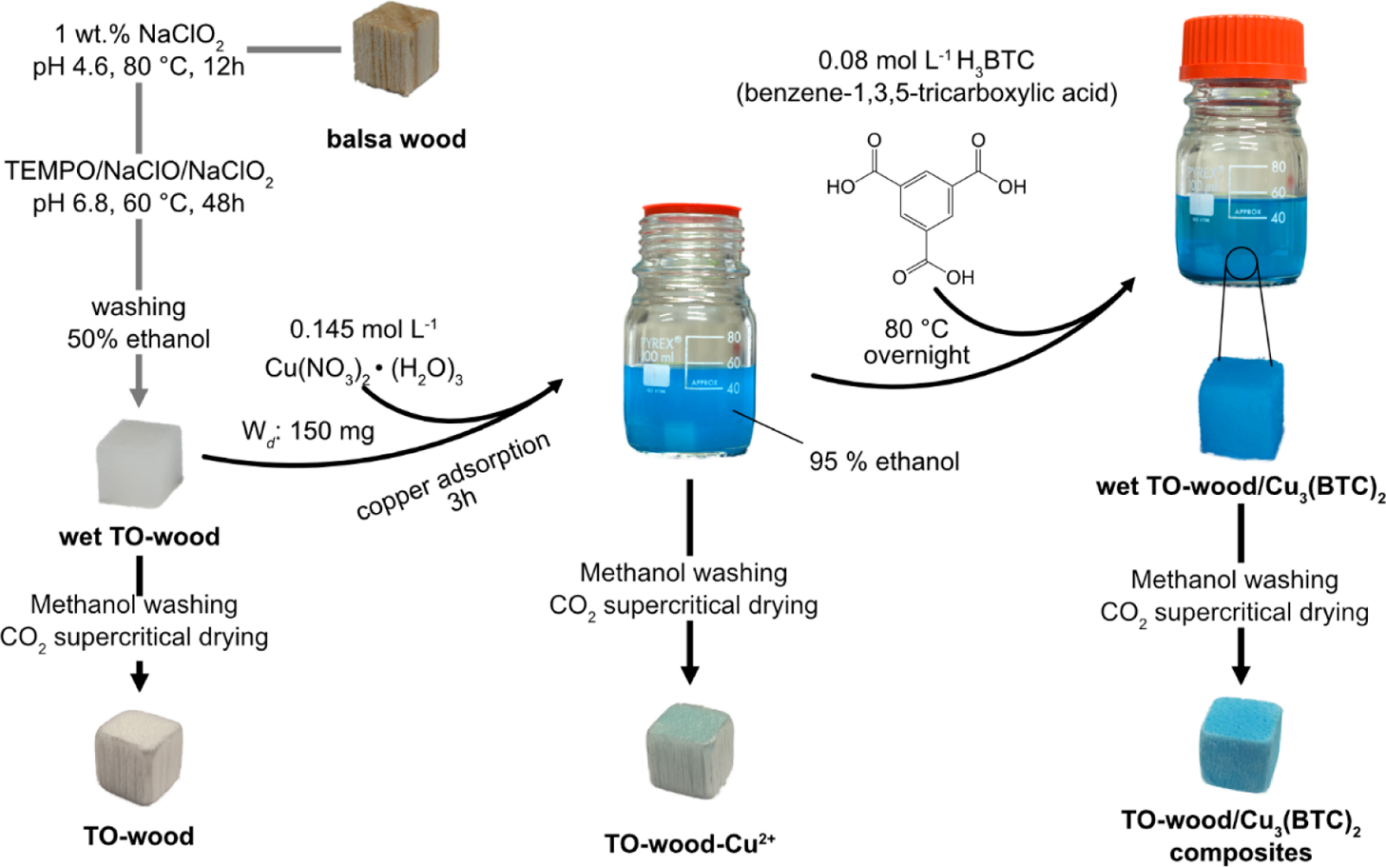
Schematic Diagram of the Synthesis Procedure for the Foam-like TO-Wood/MOF Composite Using Copper Benzene-1,3,5-tricarboxylate [Cu_3_(BTC)_2_]

As shown in Scheme 1, a balsa wood block with a dimension of 10 × 10 × 10 mm^3^ was delignified with 1 wt % sodium chlorite in sodium acetate buffer (pH 4.6) at 80 °C for 12 h. The delignified wood was then oxidized with a TEMPO/NaClO_2_/NaClO system at pH 6.8 for 48 h following the method reported in our previous work.^31^ After TEMPO-mediated oxidation, the wood blocks were further washed in an ethanol/water mixture (1:1, v/v) to remove residue chemicals and minimize swelling in water, producing the TO-wood.

### 2.3 Synthesis of TO-Wood/MOF Composites

The in situ synthesis of copper benzene-1,3,5-tricarboxylate [Cu_3_(BTC)_2_] in TO-wood was carried out in one pot (Scheme 1). The wet TO-wood samples (150 mg dry mass) were preincubated in 50 mL of 95% (v/v) ethanol containing 0.145 mol L^–1^ Cu(NO_3_)_2_·(H_2_O)_3_ for 3 h to allow the adsorption of the Cu^2+^ ion. Subsequently, the organic ligand (H_3_BTC, 0.08 mol L^–1^) was added into the incubation solution and kept at 80 °C overnight to produce the TO-wood/Cu_3_(BTC)_2_ composite. The molar ratio between Cu^2+^ and H_3_BTC and their concentration in ethanol were adopted from a previously reported synthesis method for TO-CNF/MOF aerogel.^27^ The total reaction volume was chosen to ensure that the wood samples were completely submerged in ethanol in order to achieve a rather homogeneous growth of MOFs inside the wood template. Thus, an excessive amount of Cu^2+^ (more than 50 times higher than the amount of carboxylate in TO-wood) was applied. Following the above procedure and strategy, 0.2 mol L^–1^ Al(NO_3_)_3_ and 0.2 mol L^–1^ H_3_BTC were used to prepare the TO-wood/aluminum benzene-1,3,5-tricarboxylate (AlBTC) composite.^18^ For the synthesis of TO-wood/zinc 2-methylimidazolate [Zn(MeIm)_2_] composite, 0.08 mol L^–1^ Zn(NO_3_)_2_·(H_2_O)_6_ and 1.6 mol L^–1^ 2-methylimidazole were used for the synthesis.^24^ The above as-prepared TO-wood/MOF composites were washed thoroughly with methanol and dried with supercritical CO_2_ to obtain the respective composites. Delignified wood/MOF composites were also prepared using the same procedure for comparison. For neat MOF synthesis, an identical method was used in the absence of TO-wood. After the synthesis, suspensions of neat MOFs were washed with methanol several times to remove unreacted chemicals and then dried under vacuum.

### 2.4 Characterizations

The microstructure of the composites was studied by using field emission-scanning electron microscopy (FE-SEM, S-4800, Hitachi, Japan). The cross section perpendicular to the fiber axial direction was trimmed with a sliding microtome (Leica SM2010 R) prior to synthesis for ease of observation. The cross section parallel to the fiber axial direction was observed on the interior of the peeled open TO-wood/MOF composites. Fourier transform infrared (FT-IR) spectra were recorded on a Spectrum 100 FT-IR Spectrometer (PerkinElmer, USA). X-ray diffraction (XRD) patterns were obtained on a PANalytical X’Pert PRO powder diffractometer (Malvern Panalytical, UK) equipped with a Cu Kα source. N_2_ physisorption test was carried out on 3Flex (Micromeritics, USA). The specific surface area was measured from the adsorption isotherm in the relative pressure range between 0.01 and 0.2 according to the Brunauer–Emmett–Teller (BET) method. The mass contents (wt %) of copper, aluminum, and zinc in the composites were measured with an inductively coupled plasma–optical emission spectroscopy (ICP–OES) method by a Thermo Scientific iCAP 600 series instrument. Prior to ICP–OES measurements, 0.1 g of dry powder of each sample was hydrolyzed with 72% (w/w) H_2_SO_4_ assisted with an autoclave. Typical wavelengths were used to determine the concentration of Cu: 204.3 nm, 219.9 nm, and 224.7 nm; Al: 308.2, 394.4, and 396.1 nm; and Zn: 202.5, 206.2, and 213.8 nm. The average concentration obtained at different wavelengths was taken for the evaluation of MOF loading contents in the composites. Thermal stability of dried neat MOFs and the TO-wood/MOF composites was measured on a Mettler Toledo TGA/DSC1 (Switzerland). Samples were heated from 50 to 800 °C under a nitrogen atmosphere at a heating rate of 10 °C min^–1^. Gravimetric CO_2_ adsorption capacity and temperature swing cyclic CO_2_ adsorption/desorption test were carried out on a thermogravimetric analysis (TGA) instrument (Discovery TGA, TA instruments Co. Ltd., America) equipped with both CO_2_ and N_2_ gas tanks at atmospheric pressure. The sample was first outgassed under a N_2_ flow at 105 °C for 1 h to drive out adsorbed CO_2_ and then cooled down to 25 °C. The CO_2_ adsorption process was then carried out in a CO_2_ flow at 25 °C for 150 min. Cyclic adsorption/desorption was performed by repeating the abovementioned two steps. The compression test was performed on a universal mechanical tester (Instron-5566, Instron, USA) equipped with a 10 kN loading cell at a strain rate of 10% min^–1^, 23 °C, 50% relative humidity. Elastic modulus was determined from the initial linear deformation region of stress–strain curves.

## 3 Results and Discussion

### 3.1 Synthesis of the TO-Wood/MOF Composites

**Figure 1:**
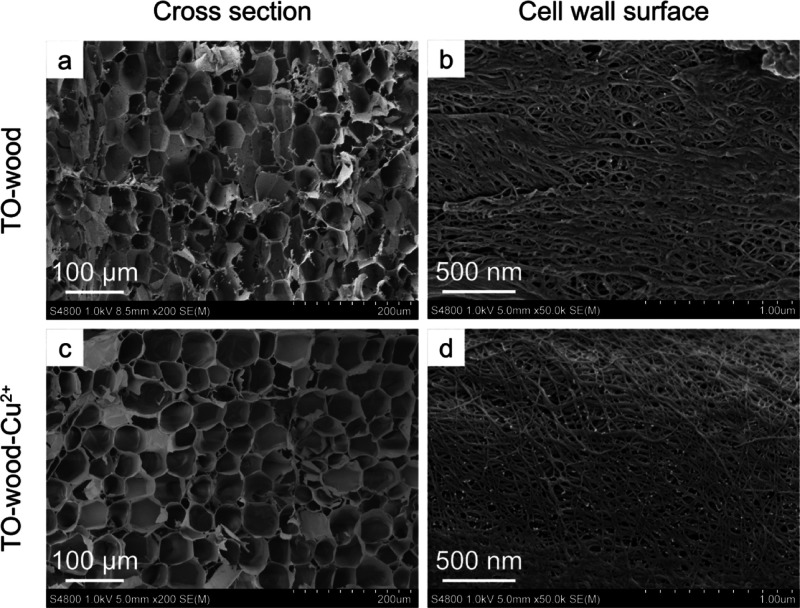
FE-SEM micrographs of the surfaces perpendicular (cross section) and parallel (cell wall surface) to the fiber axial direction for (a,b) TO-wood and (c,d) TO-wood-Cu^2+^.

**Figure 2:**
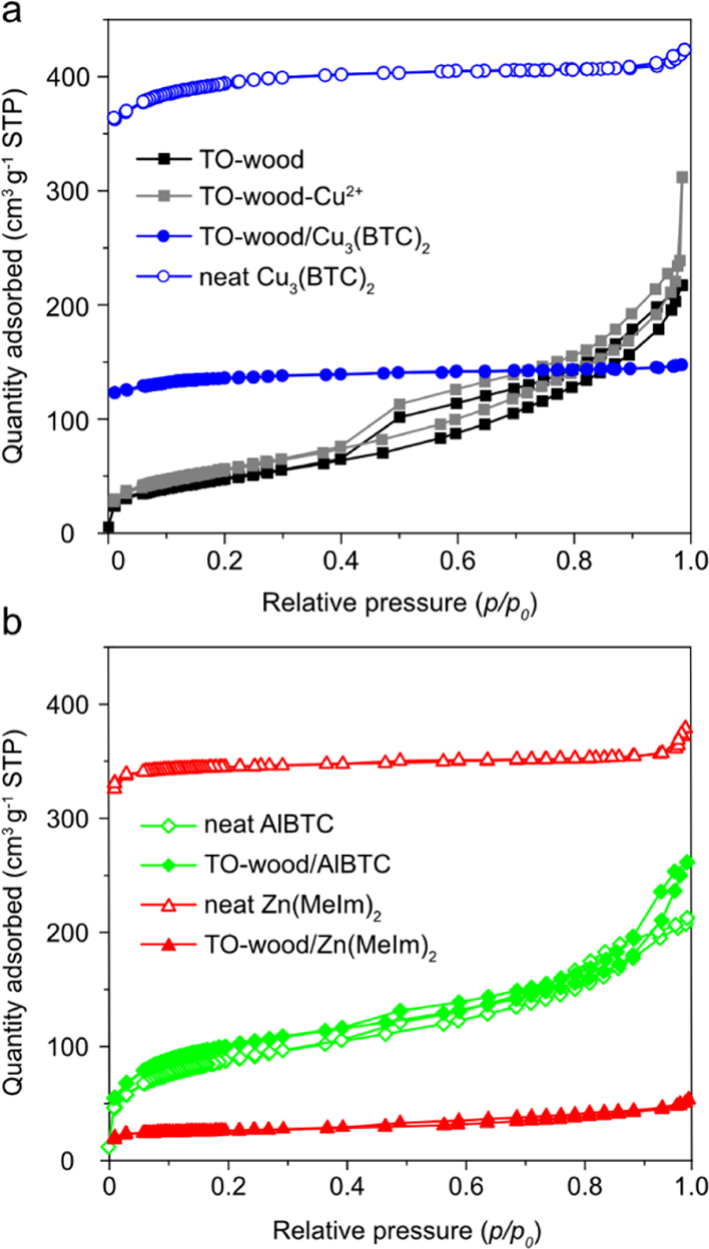
N_2_ adsorption/desorption isotherms of (a) TO-wood, TO-wood adsorbed with Cu^2+^ (TO-wood–Cu^2+^), TO-wood/Cu_3_(BTC)_2_ composite, and neat Cu_3_(BTC)_2_ and (b) neat AlBTC, TO-wood/AlBTC composite, neat Zn(MeIm)_2_, and TO-wood/Zn(MeIm)_2_ composite samples.

Through TEMPO-mediated oxidation in neutral condition, C6 hydroxyls on the surface of cellulose microfibrils in the delignified wood cell wall were selectively oxidized to carboxyl groups. To avoid extensive fibrillation during the washing step and maintain the structural integrity of the natural wood structure, the TO-wood sample was washed in an ethanol/water mixture (1:1. v/v) after the TEMPO oxidation. The content of carboxyl groups in the TO-wood was 0.66 mmol g^–1^ as determined by conductometric titration. FE-SEM revealed that the cross-sectional surface perpendicular to the fiber axial direction of TO-wood showed a honeycomb-like cellular structure similar to native balsa wood, indicating good structural integrity (Figure 1a).^32^ The hexagonal cells were slightly transformed into a round shape due to the reduced cell wall rigidity.^31^ Mesopores (2–50 nm) and macropores (>50 nm) were observed in the cell wall of TO-wood (Figure 1b). N_2_ adsorption/desorption isotherms of TO-wood showed a combination of type II and IV isotherms,^33^ with a type H3 hysteresis loop and no limiting adsorption at high *p*/*p*_0_ (Figure 2a), indicating the presence of both macro- and mesoporous structures with slit-like pores, similar to the TO-CNF/silica aerogel.^34^ The BET specific surface area (*S*_BET_) of the TO-wood was 172 m^2^ g^–1^ (Table S1, Supporting Information), about 30% lower than that reported in our previous work (249 m^2^ g^–1^).^31^ This is due to the lack of extensive washing step with water, which limited the separation of individualized cellulose microfibrils and swelling of the cell wall.

The in situ synthesis of MOFs using the wood cell wall as the template was carried out in one pot, and TO-wood was first incubated with Cu^2+^ to form the TO-wood–Cu^2+^ complex (TO-wood–Cu^2+^) through chelation. To characterize the TO-wood adsorbed with Cu^2+^, the sample was collected, washed with methanol, and dried by supercritical drying. The adsorbed amount of Cu^2+^ in TO-wood was 1.23 mmol/g, as measured by ICP–OES. This value is higher than the carboxylate content of TO-wood, indicating the unspecific binding of Cu^2+^ ion to cellulose, as reported previously.^35^ The ionic crosslinking of carboxylated cellulose microfibrils in TO-wood cell wall with divalent Cu^2+^ has led to a well-preserved native cellular structure (Figure 1c). Interconnection of microfibril bundles through thin fibrils was observed from the FE-SEM micrograph of the cell wall surface (Figure 1d). The *S*_BET_ of the TO-wood–Cu^2+^ sample was increased to 197 m^2^ g^–1^ (Table S1) with the mesoporous type IV isotherms preserved (Figure 2a). This is due to the introduction of multivalent ions that strengthened the interfibrillar interaction between cellulose microfibrils, and thus, aggregation of cellulose microfibrils and collapse of the cellulose network during supercritical CO_2_ drying were minimized. A similar effect was also reported for the TO-CNF network, in which the *S*_BET_ of TO-CNF xerogel increased from 340 to 410 m^2^ g^–1^ after crosslinking with Al^3+^.^36^

The never-dried TO-wood was preincubated with Cu^2+^ for 3 h, and the organic ligand H_3_BTC was then added in the same pot and kept at 80 °C overnight. Thus, Cu_3_(BTC)_2_ was synthesized in situ inside the TO-wood structure. After washing with methanol and drying with supercritical CO_2_, the foam-like TO-wood/Cu_3_(BTC)_2_ composite with an uniform turquoise color intrinsic to Cu_3_(BTC)_2_^37^ was obtained. The loading of Cu_3_(BTC)_2_ in the composite was 44.2 wt %, as calculated from the copper mass content measured by ICP–OES. The N_2_ adsorption/desorption isotherm of the composite (Figure 2a) showed a typical type I isotherm for microporous solid with small external surface,^33^ which was contributed by the highly microporous Cu_3_(BTC)_2_. Neat Cu_3_(BTC)_2_ exhibited a high *S*_BET_ of 1368 m^2^ g^–1^ (Table S1). After the in situ growth of Cu_3_(BTC)_2_ in TO-wood, a high *S*_BET_ value of 471 m^2^ g^–1^ was obtained for the TO-wood/Cu_3_(BTC)_2_ composite. As a comparison, the delignified wood/Cu_3_(BTC)_2_ composite showed a *S*_BET_ value of 136 m^2^ g^–1^ (Figure S1, Supporting Information) due to the much lower loading of Cu_3_(BTC)_2_ crystals (10.0 wt %) (Table S1).

In addition to Cu^2+^, the carboxyl groups in the TO-wood template can easily chelate with a series of multivalent ions including Al^3+^, Pb^2+^, Ba^2+^, Mg^2+^, Ca^2+^, Fe^3+^, Zn^2+^, and so forth*.*^27,38−40^ Thus, besides Cu_3_(BTC)_2_, in situ syntheses of Zn(MeIm)_2_ and AlBTC in TO-wood were also successfully performed via a similar procedure using different metal salts and organic ligands.

### 3.2 Structure of the TO-Wood/MOF Composites

**Figure 3:**
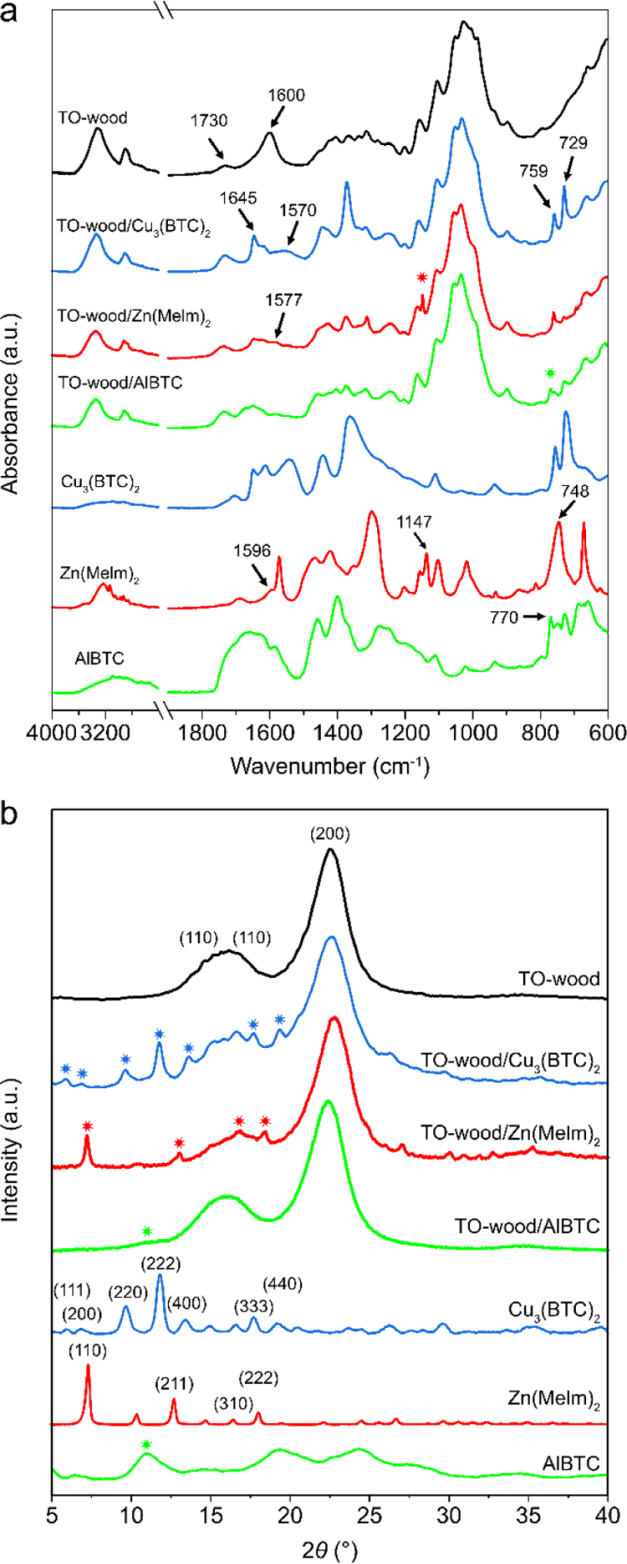
(a) FTIR spectra and (b) XRD patterns of neat MOFs, neat TO-wood, and various TO-wood/MOF composites.

The FT-IR spectra of TO-wood/Cu_3_(BTC)_2_, TO-wood/Zn(MeIm)_2_, TO-wood/AlBTC composites, neat TO-wood, and corresponding neat MOFs are compared in Figure 3a. All TO-wood/MOF composites showed an absorption peak of C=O stretching vibration mode at 1730 cm^–1^ originated from the hemicellulose in TO-wood.^31^ The band appeared at 1645 cm^–1^ in all three composites can be attributed to the following: (1) the asymmetric stretching vibration of carboxylate from BTC chelating with Cu^2+^ and Al^3+^ or (2) the C=C stretching vibration mode of the imidazole ring in Zn(MeIm)_2_, which were identified in the spectra of neat MOFs but not in the spectrum of neat TO-wood.^41−43^ The symmetric vibration peak of the carboxyl group of TO-wood in the carboxylate form shifted from 1605 cm^–1^ in neat TO-wood to around 1580–1570 cm^–1^ in the composite due to its chelation with multivalent metal ions.^24,44,45^ In addition, peaks at 729 and 759 cm^–1^ in all three TO-wood/MOF composites were assigned to the out-of-plane bending vibrations of the ring structure in either BTC or 2-methylimidazole, which were also observed for neat MOFs.^42,46^ Besides, the peak at 1147 cm^–1^ was attributed to the ring C–N stretching that is associated with 2-methylimidazole in Zn(MeIm)_2_.^47^ A unique band at 770 cm^–1^ emerged in the IR spectrum of TO-wood/AlBTC was related to the formation of the Al^3+^–COO^–^ chelation complex.^28,48^

The successful synthesis of MOFs was also confirmed by XRD analysis. Both neat TO-wood and TO-wood/MOF composites showed two broad peaks centered at 2θ = 14.8–16.8 and 22.5° (Figure 3b), corresponding to the diffraction of (11̅0), (110), and (200) planes of cellulose I crystals, respectively. In addition, peaks at 2θ = 5.9, 6.8, 9.7, 11.9, 13.8, 17.8, and 19.4° in TO-wood/Cu_3_(BTC)_2_ and neat Cu_3_(BTC)_2_ (Figure 3b) were the characteristic peaks of regular (111), (200), (220), (222), (400), (333), and (440) planes of Cu_3_(BTC)_2_, respectively.^24,49,50^ Peaks at 2θ = 7.2, 13.0, 16.8, and 18.4° that can be attributed to (110), (211), (310), and (222) planes of Zn(MeIm)_2_ were also identified for both TO-wood/Zn(MeIm)_2_ and neat Zn(MeIm)_2_.^24,27^ The characteristic peaks of AlBTC were difficult to be identified in TO-wood/AlBTC except for the weak and broad peak at 2θ = 11.0°. This was due to the large structural diversification typical for micro- and mesoporous AlBTC, showing broad and weak diffraction patterns for neat AlBTC.^18^ A similar XRD pattern has been previously reported when the synthesis of AlBTC was carried out in the presence of ethanol, where mainly the MIL-100 phase was formed.^51−53^ Compared to the simulated XRD pattern of MIL-100 (Figure S2, Supporting Information), the major broad peaks in the patterns of as-synthesized AlBTC and TO-wood/AlBTC composite were shown to closely relate to MIL-100 crystals. These results suggested that the chelation of carboxyl groups of TO-wood with multivalent ions substantially enhanced the nucleation and in situ growth of MOFs homogeneously inside TO-wood.^28^

**Figure 4:**
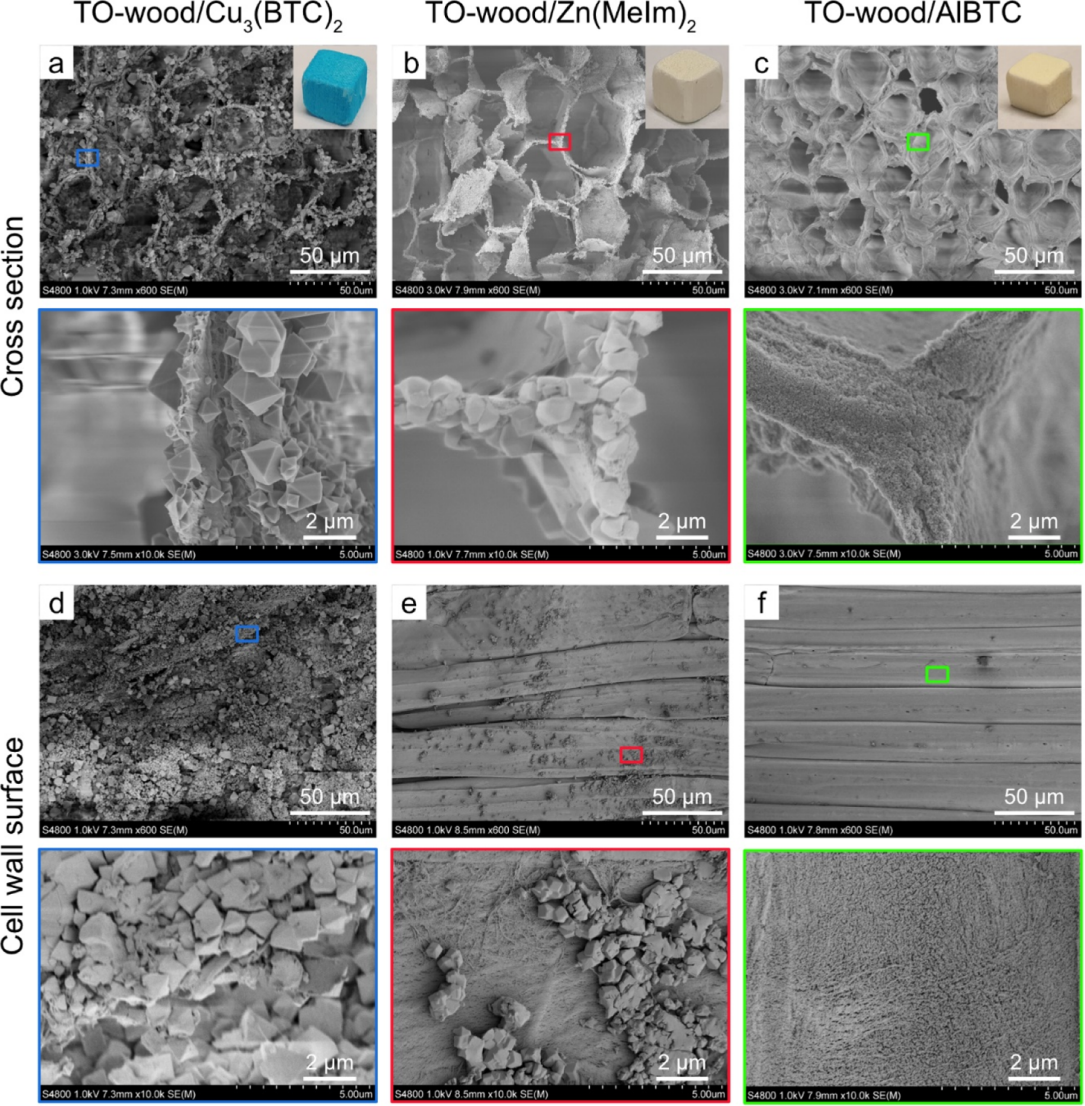
FE-SEM micrographs of low (×600) and high (×10k) magnifications showing the cellular structure and cell wall corner region in the cross-sectional surfaces perpendicular to the fiber direction and the cell wall surfaces parallel to the fiber axial direction for (a,d) TO-wood/Cu_3_(BTC)_2_, (b,e) TO-wood/Zn(MeIm)_2_, and (c,f) TO-wood/AlBTC composites, respectively. The inset photographs show the physical appearance of the composites.

Indeed, all three types of MOFs were homogeneously synthesized inside the TO-wood cell wall, leaving the lumen spaces empty for efficient mass conduction, as observed from the cross-sectional surfaces perpendicular to the fiber axial direction of the composites by FE-SEM (Figure 4a–c). The major difference was the localization and distribution of different MOF crystals due to their sizes. Image analysis revealed that the crystal sizes of Cu_3_(BTC)_2_, Zn(MeIm)_2_, and AlBTC were in the range of 0.5–5 μm, 0.5–2 μm, and 20–100 nm, respectively (Figure S3, Supporting Information). The Cu_3_(BTC)_2_ crystals were found in both inner lumen surface and intercellular region (middle lamella) (Figure 4a). The Zn(MeIm)_2_ crystals were embedded partially in the inner lumen cell wall (Figure 4b). On the other hand, the AlBTC crystals were much smaller and mainly in the form of agglomerated conformation (Figure 4c). The TO-wood cell wall was monolithically and homogeneously covered with nanoscale AlBTC crystals.

The distribution of MOFs within the wood structure was further characterized on interior surfaces of composites parallel to the fiber direction. The samples were peeled to expose the cell wall surface of the fiber cells inside the composites and observed by FE-SEM (Figure 4d–f). The Cu_3_(BTC)_2_ crystals were grown on the entire surface of fiber cells of TO-wood (Figure 4d). FE-SEM micrograph with higher magnification for the cross section of the TO-wood/Cu_3_(BTC)_2_ composite revealed that cellulose microfibrils were attached on the surface of Cu_3_(BTC)_2_ crystals that were grown inside the cell wall (Figure S4, Supporting Information), indicating the coordination between TO-wood and Cu_3_(BTC)_2_.^28^ The distribution of Zn(MeIm)_2_ crystals was rather heterogeneous and scattered along the fiber cell surface with large cell wall surface areas exposed (Figure 4e). The particle size of AlBTC crystals was below 100 nm and AlBTC nanocrystals were embedded in the cellulose microfibril network (Figure 4f). The reason for high loading of Cu_3_(BTC)_2_ (44.2 wt %) in TO-wood can be attributed to the higher affinity of Cu^2+^ to the C6 carboxyl groups of TO-CNFs compared to Zn^2+^.^35^ Therefore, more metal ions were adsorbed and served as nucleation sites for the synthesis of MOFs. Indeed, the adsorbed amount of Zn^2+^ to TO-wood was 0.31 mmol/g, as measured by ICP–OES, much lower than that for Cu^2+^ (1.23 mmol/g). Thus, it resulted in a lower loading of Zn(MeIm)_2_ in the composite (11.3 wt %). Interestingly, the adsorbed amount of Al^3+^ to TO-wood was 0.30 mmol/g, but the loading of AlBTC crystals in the composites was remarkably high (42.7 wt %). This is probably due to the reason that an excess amount of AlBTC was synthesized in the bulk solution and trapped inside the cell wall because of its small particle size (20–100 nm). The TO-wood/Zn(MeIm)_2_ and TO-wood/AlBTC composites also showed enhanced BET surface areas of 92 and 361 m^2^ g^–1^ (Figure 2b and Table S1) as compared to 37 and 38 m^2^ g^–1^ for delignified wood/Zn(MeIm)_2_ and delignified wood/AlBTC (Figure S1 and Table S1), respectively. The higher loading of MOFs and large surface area thus can endow TO-wood/MOF composites with competitive CO_2_ adsorption performance.

**Figure 5:**
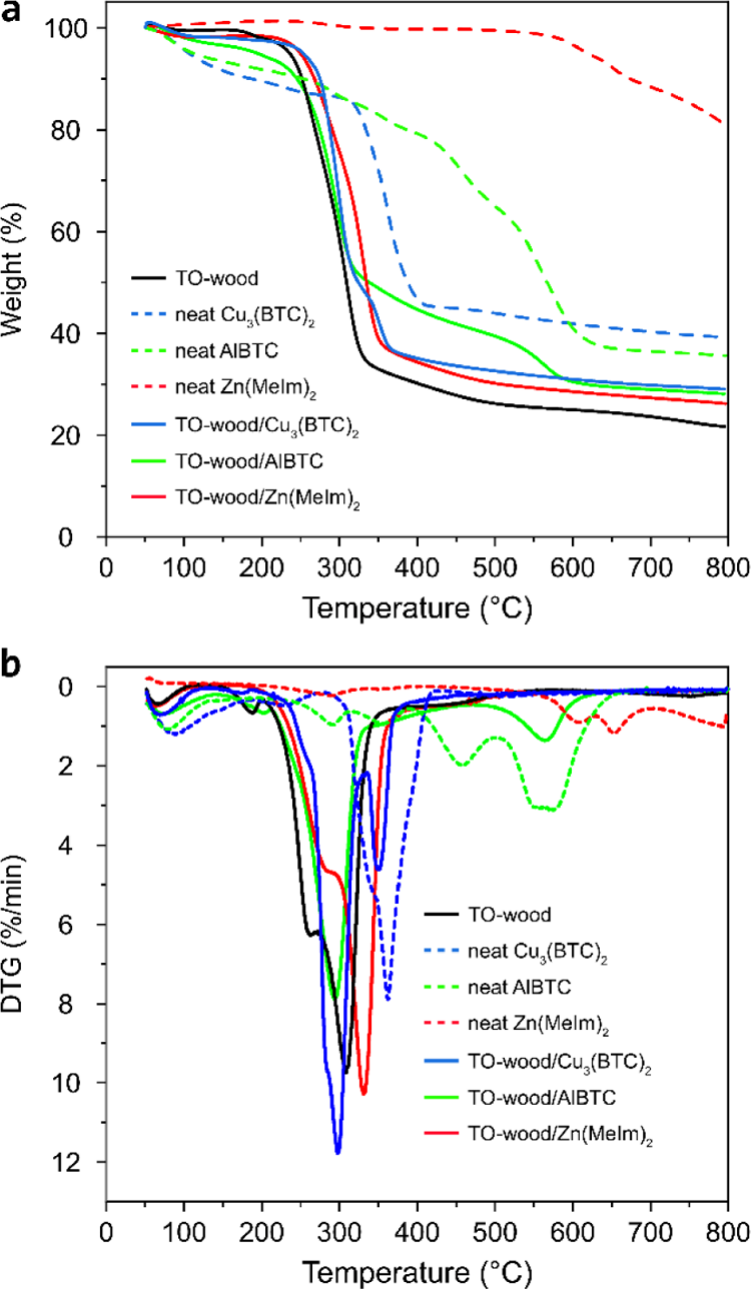
(a) Thermogravimetric curves and (b) corresponding first derivative weight loss curves of neat MOFs, TO-wood, and the TO-wood/MOF composites.

The decomposition behavior of the TO-wood/MOF composites was studied by TGA. As shown in Figure 5, the decomposition of TO-wood started at around 240 °C due to the thermal degradation of cellulose. The structural decomposition of neat Cu_3_(BTC)_2_ took place at a higher temperature, in the range of 320–400 °C, after a first stage of water evaporation (∼100 °C) and a second stage of decomposition of low-quality crystals (∼220 °C).^54^ The TO-wood/Cu_3_(BTC)_2_ composite showed an enhanced thermal stability as compared to TO-wood. It showed two main decomposition stages at 270–330 and 330–370 °C due to the decomposition of TO-wood and Cu_3_(BTC)_2_, respectively. Similarly, the incorporation of Zn(MeIm)_2_ and AlBTC also improved the thermal stability of TO-wood. Neat Zn(MeIm)_2_ is highly thermally stable and showed a profile of nearly constant weight up to 570 °C. The in situ growth of Zn(MeIm)_2_ in TO-wood led to the increase of the initial decomposition temperatures of TO-wood/Zn(MeIm)_2_ to 250 °C. Neat AlBTC showed a multistage decomposition profile in the range of 275–650 °C. Compared to TO-wood, the decomposition of TO-wood/AlBTC started at a higher temperature of 255 °C, with a second decomposition stage in the range of 320–620 °C due to the high loading of AlBTC in the composite.

### 3.3 CO_2_ Adsorption/Desorption Performance

**Figure 6:**
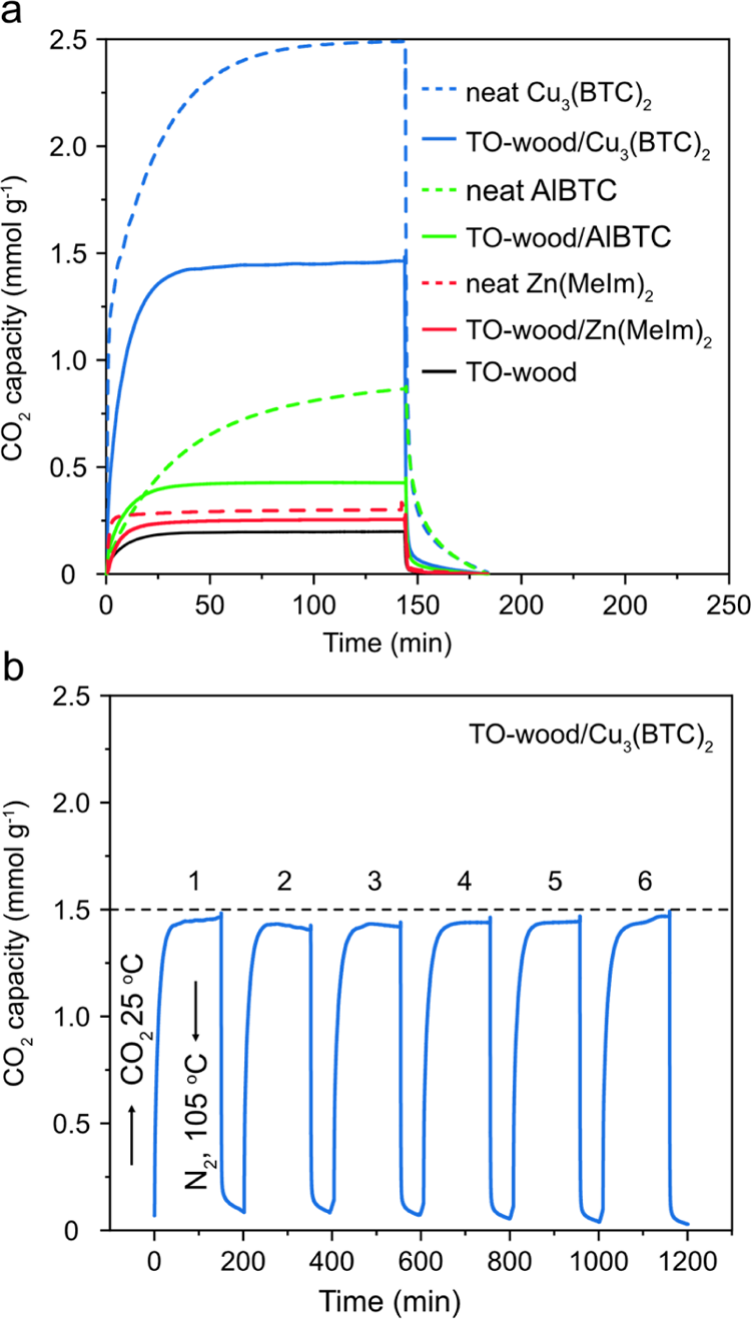
(a) CO_2_ adsorption/desorption isotherms of neat MOFs, neat TO-wood, and various TO-wood/MOF composites. (b) Temperature swing cyclic CO_2_ adsorption/desorption isotherms of the TO-wood/Cu_3_(BTC)_2_ composite (adsorption under CO_2_ at 25 °C and desorption under N_2_ at 105 °C).

CO_2_ adsorption/desorption isotherms of neat wood template, neat MOFs, and TO-wood/MOF composites were recorded using TGA equipped with gas tanks containing pure dry CO_2_ and N_2_, respectively. The adsorption of CO_2_ was performed at 25 °C and atmospheric pressure after an initial drying with N_2_ at 105 °C. Moreover, desorption was conducted right after adsorption with a constant flow of N_2_ at 105 °C. Neat Cu_3_(BTC)_2_ MOFs exhibited the highest CO_2_ adsorption capacity of 2.49 mmol g^–1^, which is equivalent to 11.0 wt % CO_2_ uptake (Figure 6a). This is consistent with the previously reported result that the maximum CO_2_ adsorption to Cu_3_(BTC)_2_ at 295 K and 0.1 MPa was 2.46 mmol g^–1^ through physical trapping of CO_2_ molecules.^55^ Neat TO-wood also showed a CO_2_ adsorption capacity of 0.2 mmol/g, which was caused by the binding of CO_2_ to the carboxyl group through dipolar interaction.^56^ As a result of in situ growth of MOFs inside TO-wood, the TO-wood/Cu_3_(BTC)_2_ composite showed a high CO_2_ adsorption capacity of 1.46 mmol g^–1^. Cu_3_(BTC)_2_ contains coordinatively unsaturated metal sites for CO_2_ adsorption, which is greatly beneficial when used for cyclic CO_2_ adsorption.^57^ This enables Cu_3_(BTC)_2_ to be the favored MOF material for CO_2_ capture and storage. On the contrary, the CO_2_ adsorption capacities of neat Zn(MeIm)_2_ and AlBTC at 25 °C and atmospheric pressure were 0.30 and 0.87 mmol g^–1^, about 8 and 3 times lower than that of neat Cu_3_(BTC)_2_. These results were in line with the literature result where ZIF-8 showed CO_2_ capacity around 0.4 mmol g^–1^ at 25 °C and 1 bar.^58^ Therefore, the TO-wood/Zn(MeIm)_2_ and To-wood/AlBTC composites showed lower CO_2_ adsorption capacities of 0.25 and 0.43 mmol g^–1^, respectively.

The CO_2_ adsorption capacities and conditions of several wood- or cellulose-based sorbents are summarized in Table 1. At ambient condition, the CO_2_ adsorption capacity of TO-wood/Cu_3_(BTC)_2_ in this work is superior to that of the wood- or cellulose-based CO_2_ sorbents loaded with PEI,^59^ acetylated CNCs,^60^ and zeolite^61^ and is comparable to that of the APTES-grafted TO-CNFs/silica aerogels.^34^ Although the TO-CNFs/PEI foam showed a high CO_2_ adsorption capacity of 2.22 mmol g^–1^ at 80% RH, contribution from water is negligible. The same material tested at a lower humidity of 20% RH showed only one-fourth of its maximum capacity.^62^ Moreover, CO_2_ sorption sites, primarily amine groups, in amine-grafted sorbents are often gradually deactivated after the cyclic regeneration process, that is, thermal-driven desorption at higher temperature.^62−64^ As a consequence, the maximum CO_2_ capacity of TO-CNFs/PEI foam decreased 27% after five cycles of adsorption/desorption at 25 and 85 °C.^62^ 7% capacity loss after 10 cycles was also reported on PEI crosslinked cellulose triacetate aerogel when desorption was conducted at 105 °C.^65^ MOFs are generally thought as thermally stable with thermal decomposition temperatures higher than 300 °C.^66^ To have a better understanding of the reversibility and thermal stability of CO_2_ adsorption by the TO-wood/Cu_3_(BTC)_2_ composite, cyclic CO_2_ adsorption/desorption test was performed. The CO_2_ adsorption capacity during the first cycle was 1.46 mmol g^–1^. After six cycles of adsorption/desorption at 25 and 105 °C, 1.47 mmol g^–1^ CO_2_ capacity was measured (Figure 6b), demonstrating the excellent multicycle durability.

**Table 1:** CO_2_ Adsorption Capacity of Various Wood- or Cellulose-Based CO_2_ Sorbents

	capacity mmol g–^1^	pressure	temperature (°C)	humidity
TO-wood/Cu_3_(BTC)_2_	1.46	atmospheric pressure	25	dry
delignified wood/PEI^59^	1.11	atmospheric pressure	25	dry
CNFs/acetylated CNCs^60^	1.14	101 kPa	0	dry
TO-CNFs/gelatin/zeolite^61^	∼1.3	750 mmHg	35	dry
TO-CNFs/silica/APTES^34^	1.49	atmospheric pressure	25	dry
TO-CNFs/PEI^62^	2.22	ambient	25	80% RH
TO-CNFs/PEI^62^	0.5	ambient	25	20% RH

### 3.4 Compressive Mechanical Properties

**Figure 7:**
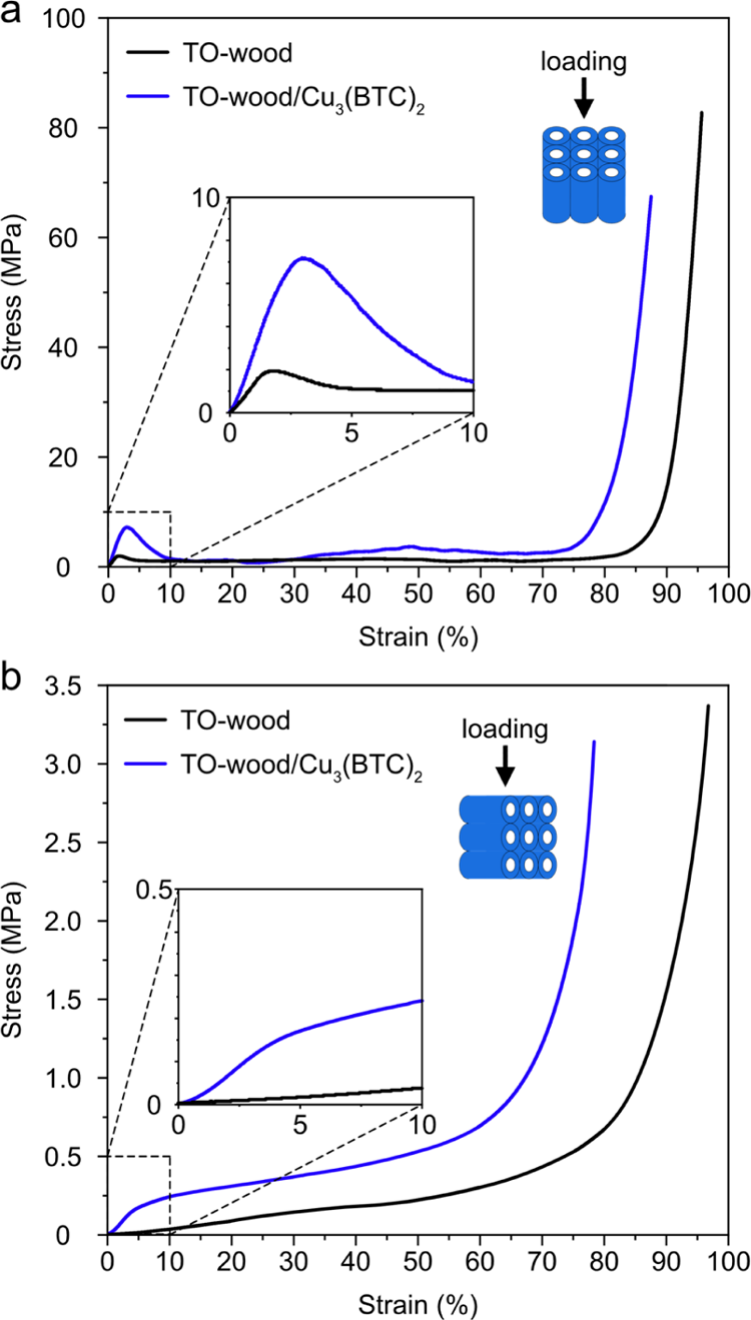
Typical compressive stress–strain curves of the TO-wood/Cu_3_(BTC)_2_ composite and TO-wood with the loading (a) parallel to the fiber axial direction, i.e., longitudinal direction and (b) perpendicular to the fiber axial direction, i.e., transverse direction. The insets are the corresponding stress–strain curves in the strain range of 0–10%.

Typical compressive stress–strain curves of the TO-wood/Cu_3_(BTC)_2_ composites and TO-wood are shown in Figure 7. The TO-wood/Cu_3_(BTC)_2_ composite and TO-wood had low densities of 107.4 ± 5.5 and 61.1 ± 4.3 kg m^–3^, respectively. When the loading was parallel to the fiber axial (longitudinal) direction, both TO-wood/Cu_3_(BTC)_2_ composite and TO-wood showed initial linear elastic deformation with rapid increase of compressive stress at compressive strain lower than 2% (Figure 7a). After the yielding point, both materials showed plastic deformation plateaus at moderate strain before entering a final densification phase, during which the stress increased exponentially. The deformability of TO-wood/Cu_3_(BTC)_2_ was considerably better than those of inorganic monolithic sorbents since no catastrophic failure of materials was observed, demonstrating good mechanical integrity. Compared to neat TO-wood, the elastic modulus of the TO-wood/Cu_3_(BTC)_2_ composite was increased from 125.4 ± 26.2 to 326.2 ± 7.5 MPa, indicating the positive effect of ionic crosslinking by Cu^2+^ on the stiffness of the TO-wood template, which was also reported on the TO-CNF hydrogel crosslinked with multivalent ions.^36^ The yield strain of the TO-wood/Cu_3_(BTC)_2_ composite was about 3.5%, higher than 2.2% for TO-wood, which is owing to the enhanced energy dissipation with the addition of MOFs. The compressive yield strength of the TO-wood/Cu_3_(BTC)_2_ composite was 7.3 ± 0.1 MPa, 3 times higher than that of the neat TO-wood (2.4 ± 0.4 MPa). This is due to (1) the crosslinking effect of Cu^2+^, which led to the improved resistance to yield the TO-wood/Cu_3_(BTC)_2_ composite, and (2) the reinforcing effect of MOFs on the TO-wood/Cu_3_(BTC)_2_ composite through a strong interfacial interaction with the TO-wood cell wall, which was also reported for the ZIF-8 reinforced wood composite.^24^

Due to the inherent anisotropy of wood, TO-wood/Cu_3_(BTC)_2_ showed different deformation behaviors in the transverse direction (Figure 7b). Both TO-wood/Cu_3_(BTC)_2_ composite and TO-wood showed good compressibility. The TO-wood/Cu_3_(BTC)_2_ composite showed a short linear elastic region up to a compressive strain of 5%, followed by a stress plateau in the range of 5–60% compressive strain, in which the cell wall was collapsed gradually.^67^ Thereafter, a rapid densification region appeared, indicating the elimination of cell wall porosity. Similar compressive stress–strain behavior was also reported for the ultralight TO-CNF/MIL-53 aerogel.^28^ The elastic modulus and yield strength of the TO-wood/Cu_3_(BTC)_2_ composite were 3.5 ± 0.4 and 0.20 ± 0.04 MPa, respectively, which are still considerably high. As a comparison, the neat TO-wood barely showed a yielding phenomenon and was gradually densified as the compressive strain increased to 90%, with a 10 times lower elastic modulus recorded at 0.36 ± 0.05 MPa.

The mechanical properties of the TO-wood/Cu_3_(BTC)_2_ composite were exceptional as compared with various monolithic CO_2_ sorbents summarized in Table 2. Although the compressive elastic modulus and yield strength of the TO-wood/Cu_3_(BTC)_2_ composite in the axial direction were *ca.* 40% of those of the delignified wood/PEI composite,^59^ the density of TO-wood/Cu_3_(BTC)_2_ was only 30% of that of delignified wood/PEI (343.6 ± 15.3 kg m^–3^). The specific elastic modulus (*E*_s_: 3034 kN m kg^–1^) and specific yield strength (σ_s_: 68 kN m kg^–1^) of TO-wood/Cu_3_(BTC)_2_ in the axial direction were remarkably high. These values were also higher than those for cellulose-based CO_2_ sorbents such as APTES-grafted TO-CNF/silica aerogel,^34^ anisotropic CNF aerogel impregnated with acetylated CNC,^60^ and anisotropic foam of TO-CNFs/gelatin/zeolite.^61^ Moreover, in comparison to MOF-loaded monolithic CO_2_ sorbents, the TO-wood/Cu_3_(BTC)_2_ composite was 2 orders of magnitude stronger than the graphene/ZIF-8 aerogel^22^ and 3D-printed MOF/clay/PVA monoliths,^19^ suggesting TO-wood as a much more robust template for structuring MOFs into a monolith than using an inorganic substrate or polymer-based binders.

**Table 2:** Compressive Mechanical Performance of Various CO_2_ Monolithic Sorbents^a^

	test direction	elastic modulus (MPa)	yield strength (MPa)	specific elastic modulus (kN m kg^–1^)	specific yield strength (kN m kg^–1^)
TO-wood/Cu_3_(BTC)_2_	axial	326.2 (7.5)	7.3 (0.1)	3034	68
TO-wood/Cu_3_(BTC)_2_	transverse	3.5 (0.4)	0.20 (0.04)	33	2
delignified wood/PEI^59^	axial	756	18	2200	52
CNF/silica aerogel^34^	isotropic	0.18	0.03	22	4
CNF/acetyl-CNCs aerogel^60^	axial	0.30	0.02	20	2
TO-CNFs/gelatin/zeolite^61^	axial	2.2		147	
Graphene/ZIF8 aerogel^22^	isotropic	0.28	0.02	12	1
PVA/clay/MOF-74(Ni)^19^	axial	12	0.48	13	0.5
PVA/clay/UTSA-16(Co)^19^	axial	25	0.55	15	0.3

a The values in parentheses are the sample standard deviations.

## 4 Conclusions

In summary, foam-like TO-wood/MOF composites were successfully prepared by the in situ synthesis of Cu_3_(BTC)_2_, Zn(MeIm)_2_, and AlBTC in a TO-wood template. The surface carboxyl group on the cellulose microfibrils in the TO-wood facilitated the interfacial coordination of multivalent metal ions and subsequent MOF nucleation and growth in the wood cell wall. The TO-wood/Cu_3_(BTC)_2_ composite had a high loading (44.2 wt %) of Cu_3_(BTC)_2_, a large BET surface area of 471 m^2^ g^–1^, and a high CO_2_ adsorption capacity of 1.46 mmol g^–1^ at 25 °C and atmospheric pressure, higher than those for the TO-wood/Zn(MeIm)_2_ and TO-wood/AlBTC composites. Besides, the TO-wood/Cu_3_(BTC)_2_ composite maintained the maximum capacity during the temperature swing cyclic CO_2_ adsorption test, demonstrating good multicycle durability. Moreover, the TO-wood/Cu_3_(BTC)_2_ composite was exceptionally strong in the longitudinal direction (fiber axial) with a remarkably high specific elastic modulus of 3034 kN m kg^–1^ and a high specific yield strength of 68 kN m kg^–1^ ever reported for solid CO_2_ sorbents. This study introduced a facile strategy to address the interfacial coordination of MOFs to the wood cell wall and significantly increased the loading of MOFs in the wood structure and therefore achieved the foam-like composites combining the versatile functionalities of MOFs and the mechanical robustness of wood. The TO-wood/MOF composites are also promising for various possible applications in environmental remediation, gas separation and purification, insulation, and catalysis and will be further investigated in the future.

## Supporting Information Available

N_2_ adsorption/desorption isotherms of delignified wood/MOF composites; comparison of XRD patterns of TO-wood/AlBTC, as-synthesized neat AlBTC, and simulated MIL-100; histograms of size distribution for the MOF particles in the composites; FE-SEM image for the cross section of the TO-wood/Cu_3_(BTC)_2_ composite at higher magnification; and MOF contents and BET surface areas of the delignified wood/MOF composites and TO-wood/MOF composites (PDF)
