# Supplementary material for: Strong Foam-like Composites from Highly Mesoporous Wood and Metal-Organic Frameworks for Efficient CO2 Capture
Source: ACS Appl Mater Interfaces. 2021 Jun 15;13(25):29949–59. doi: 10.1021/acsami.1c06637 (PMC8289243; doi:10.1021/acsami.1c06637)
Supplement: Supplementary file 1 — am1c06637_si_001.pdf [file am1c06637_si_001.pdf]

Supporting Information

Strong Foam-like Composites from Highly Mesoporous Wood and Metal Organic Frameworks for Efficient CO_2 Capture

Shennan Wang[†], *Cheng Wang*[‡], *Qi Zhou*[†,§,*]

[†] Division of Glycoscience, Department of Chemistry, School of Engineering Sciences in Chemistry, Biotechnology and Health, KTH Royal Institute of Technology, AlbaNova University Centre, Stockholm SE-106 91, Sweden

[‡] Advanced Fibro-Science, Kyoto Institute of Technology, Kyoto 606-8585, Japan

[§] Wallenberg Wood Science Center, Department of Fibre and Polymer Technology, KTH Royal Institute of Technology, Stockholm SE-100 44, Sweden

* Corresponding author, Email: qi@kth.se

This Supporting Information contains 1 Table and 4 Figures.

Table S1. MOFs contents and BET surface area of neat MOFs, wood templates, delignified wood/MOFs composites and TO-wood/MOFs composites.

	MOFs content (wt.%) [a]	S_{BET} (m^2 g^{-1})
neat $Cu_3(BTC)_2$	100	1368
neat $Zn(MeIm)_2$	100	1196
neat AlBTC	100	682
TO-wood	-	172
TO-wood-Cu^{2+}	-	197
Delignified wood/$Cu_3(BTC)_2$	10.0	136
Delignified wood/$Zn(MeIm)_2$	4.1	37
Delignified wood/AlBTC	3.3	38
TO-wood/$Cu_3(BTC)_2$	44.2	471
TO-wood/$Zn(MeIm)_2$	11.3	92
TO-wood/AlBTC	42.7	361

[a] Calculated based on the mass content of metal determined by using inductively coupled plasma-optical emission spectroscopy (ICP-OES).

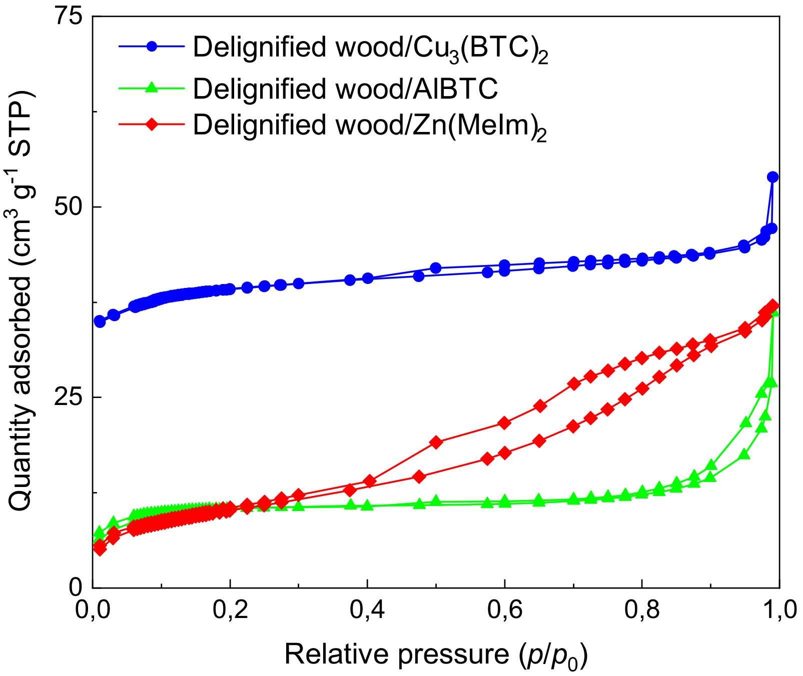

Figure S1. N_2 adsorption/desorption isotherms of the delignified wood/MOFs composites.

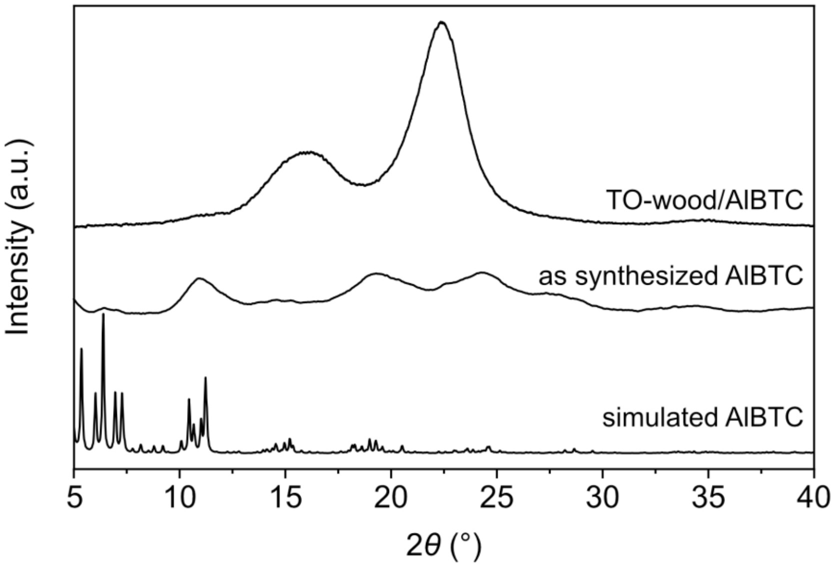

Figure S2. XRD patterns of the TO-wood/AlBTC composite, as synthesized AlBTC and simulated AlBTC (MIL-100).

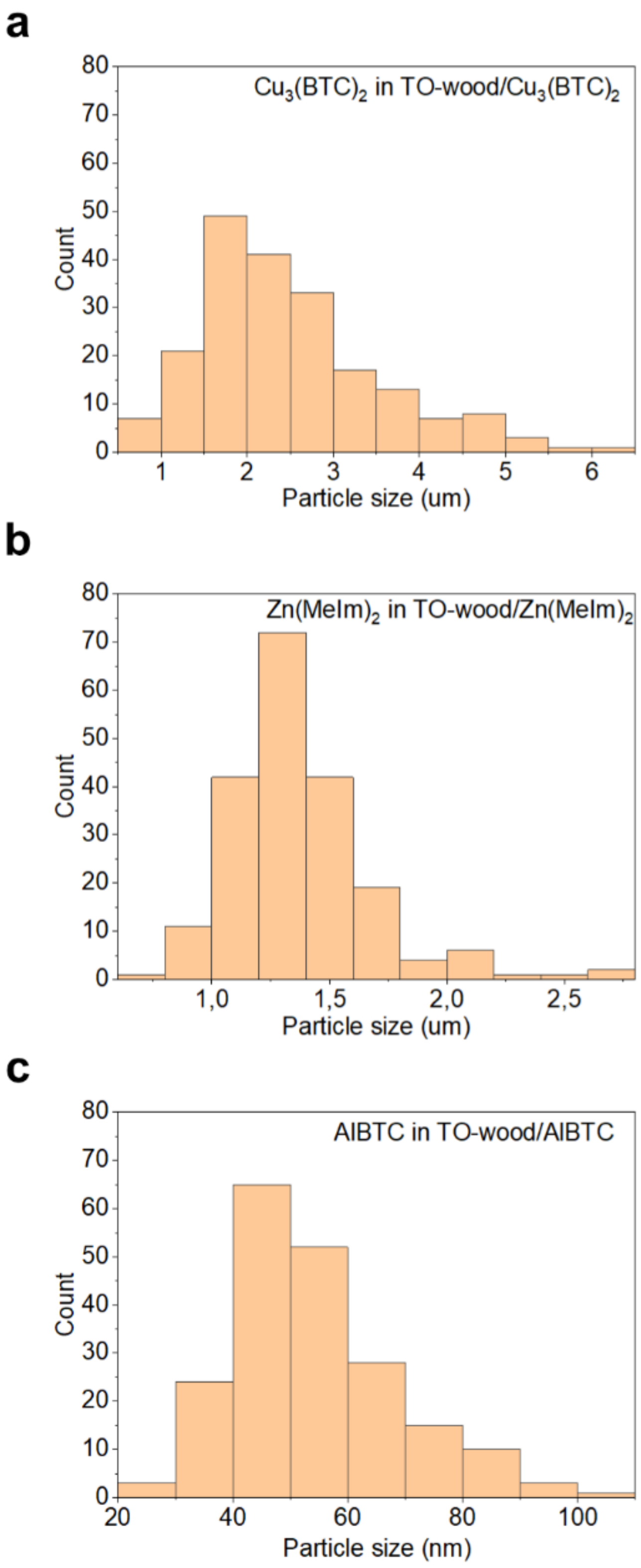

Figure S3. Histograms of size distribution for (a) $Cu_3(BTC)_2$, (b) $Zn(MeIm)_2$, and (c) AlBTC MOFs particles in the corresponding TO-wood/MOFs composites.

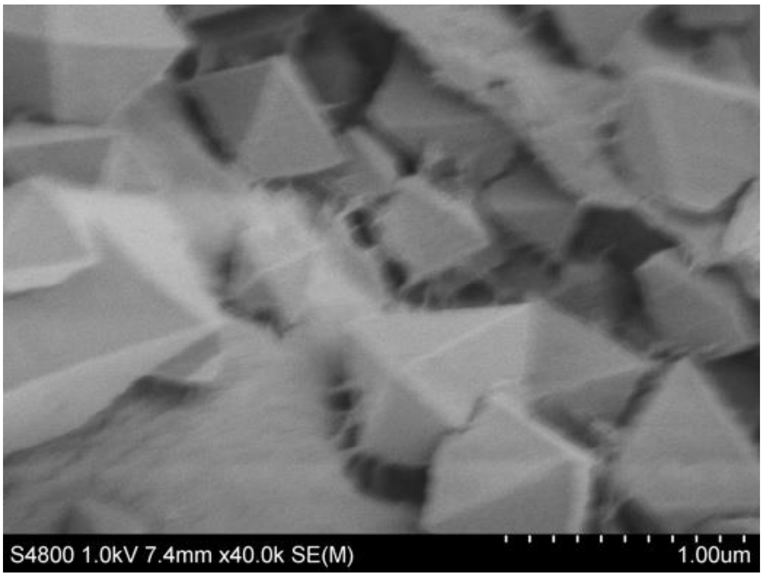

Figure S4. FE-SEM micrograph with higher magnification for cross section of the TO-wood/$Cu_3(BTC)_2$ composite at the cell wall surface reveals that cellulose microfibrils were attached on the surface of $Cu_3(BTC)_2$ crystals that were grown inside the cell wall.
